# RETRACTION: Exploring the Impact of TGF‐β Family Gene Mutations and Expression on Skin Wound Healing and Tissue Repair

**DOI:** 10.1111/iwj.70430

**Published:** 2025-03-26

## Abstract

**RETRACTION:** CuiK. , GongS. , BaiJ. , XueL. , LiX. , and WangX. , “Exploring the Impact of TGF‐β Family Gene Mutations and Expression on Skin Wound Healing and Tissue Repair,” International Wound Journal 21, no. 4 (2024): e14596, 10.1111/iwj.14596.38151761 PMC10961875

The above article, published online on 27 December 2023, in Wiley Online Library (http://onlinelibrary.wiley.com/), has been retracted by agreement between the journal Editor in Chief, Professor Keith Harding; and John Wiley & Sons Ltd. Following an investigation by the publisher, all parties have concluded that this article was accepted solely on the basis of a compromised peer review process. The editors have therefore decided to retract the article. The authors did not respond to our notice regarding the retraction.

**RETRACTION:** K. Cui , S. Gong , J. Bai , L. Xue , X. Li , and X. Wang , “Exploring the Impact of TGF‐β Family Gene Mutations and Expression on Skin Wound Healing and Tissue Repair,” International Wound Journal 21, no. 4 (2024): e14596, 10.1111/iwj.14596.38151761 PMC10961875

The above article, published online on 27 December 2023, in Wiley Online Library (http://onlinelibrary.wiley.com/), has been retracted by agreement between the journal Editor in Chief, Professor Keith Harding; and John Wiley & Sons Ltd. Following an investigation by the publisher, all parties have concluded that this article was accepted solely on the basis of a compromised peer review process. The editors have therefore decided to retract the article. The authors did not respond to our notice regarding the retraction.

